# Spatial genomics reveals cholesterol metabolism as a key factor in colorectal cancer immunotherapy resistance

**DOI:** 10.3389/fonc.2025.1549237

**Published:** 2025-03-18

**Authors:** Andrew J. Kavran, Yulong Bai, Brian Rabe, Anna Kreshock, Andrew Fisher, Yelena Cheng, Anne Lewin, Chao Dai, Matthew J. Meyer, Konstantinos J. Mavrakis, Anna Lyubetskaya, Eugene Drokhlyansky

**Affiliations:** ^1^ Mechanisms of Cancer Resistance Thematic Research Center (TRC), Bristol Myers Squibb, Cambridge, MA, United States; ^2^ Informatics and Predictive Sciences, Bristol Myers Squibb, Cambridge, MA, United States; ^3^ Translational Medicine, Bristol Myers Squibb, Cambridge, MA, United States

**Keywords:** spatial transcriptomics, cholesterol, MC38, colorectal cancer, Visium, PD-1, immunotherapy resistance, spatial genomics

## Abstract

Immune checkpoint inhibitors (ICIs) have transformed the treatment landscape across multiple cancer types achieving durable responses for a significant number of patients. Despite their success, many patients still fail to respond to ICIs or develop resistance soon after treatment. We sought to identify early treatment features associated with ICI outcome. We leveraged the MC38 syngeneic tumor model because it has variable response to ICI therapy driven by tumor intrinsic heterogeneity. ICI response was assessed based on the level of immune cell infiltration into the tumor – a well-established clinical hallmark of ICI response. We generated a spatial atlas of 48,636 transcriptome-wide spots across 16 tumors using spatial transcriptomics; given the tumors were difficult to profile, we developed an enhanced transcriptome capture protocol yielding high quality spatial data. In total, we identified 8 tumor cell subsets (*e.g.*, proliferative, inflamed, and vascularized) and 4 stroma subsets (*e.g.*, immune and fibroblast). Each tumor had orthogonal histology and bulk-RNA sequencing data, which served to validate and benchmark observations from the spatial data. Our spatial atlas revealed that increased tumor cell cholesterol regulation, synthesis, and transport were associated with a lack of ICI response. Conversely, inflammation and T cell infiltration were associated with response. We further leveraged spatially aware gene expression analysis, to demonstrate that high cholesterol synthesis by tumor cells was associated with cytotoxic CD8 T cell exclusion. Finally, we demonstrate that bulk RNA-sequencing was able to detect immune correlates of response but lacked the sensitivity to detect cholesterol synthesis as a feature of resistance.

## Introduction

Immune checkpoint inhibitors (ICIs) transformed the field of immuno-oncology and the treatment landscape for multiple cancer types, including melanoma (1), non-small cell lung cancer (2), renal cell carcinoma (3), and microsatellite instability-high (MSI-H) colorectal carcinoma (CRC) (4, 5). Antibodies targeting PD-1 (aPD-1), nivolumab and pembrolizumab, represent two of the leading ICI therapies (6). Their efficacy is associated with the presence of infiltrating CD8 T cells in the tumor, which drive tumor cell clearance when the interaction between PD1 and its ligand, PD-L1, is blocked (7–9).

Despite their success, many patients either do not respond to ICI treatments or develop resistance following initial response due to tumor cell intrinsic and extrinsic factors (10–12). This complexity is well captured using single cell and spatial genomics, (13–23) and studies to date have characterized both post-treatment human (15, 24–26) and mouse tumors (27–30) suggesting there is a progression to developing ICI therapy resistance. First, intrinsic tumor variability determines the level of initial ICI response. For example, the baseline levels of MSI status and PD-L1 expression are well-established predictive biomarkers of response (31). Next, intrinsically recalcitrant tumors develop further adaptations leading to immune evasion and suppression. These adaptations include resistance to immune surveillance through JAK/STAT pathway mutations (32), reduced antigenicity (32, 33) and metabolic changes (34–36). In complement, the recruitment and action of immunosuppressive cells (*e.g.*, myeloid-derived suppressor cells, regulatory T cells, and stromal fibroblasts) dampen immune responses by cytotoxic T cells (37–40). Here, we sought to further understand how tumor intrinsic heterogeneity shapes the initial phase of ICI response and resistance by characterizing tumors early in their pharmacodynamic response. Specifically, we leveraged spatial genomics and pathology approaches to characterize the association of tumor intrinsic heterogeneity with the level of cytotoxic T cell infiltration stimulated by short term aPD-1 antibody administration *in vivo*.

We selected the MC38 syngeneic CRC model due to its variable response to aPD-1 therapy, which enables the study of ICI response and resistance based on intrinsic heterogeneity within a single model (28, 30, 41–43). We reasoned that tumor cell intrinsic signaling heterogeneity contributes to the variable aPD-1 response phenotype making this an ideal reductionist model to dissect differences in tumor cell intrinsic signaling associated with ICI response. Additionally, we sought to evaluate the utility of spatial transcriptomics (ST) to identify resistance mechanisms relative to traditional bulk profiling methods (20, 21, 44–50).

We generated a spatial map comprising transcriptomics and histology characterization of aPD-1 early responders and non-responders. This map includes 48,636 spatially resolved, transcriptome-wide, low-bulk spots from aPD-1 and control IgG treated tumors, matched to complementary immunohistochemistry-staining (*i.e.*, CD4 and CD8) and histology (*i.e.*, H&E) analysis. These multi-modal data enabled orthogonal validation of aPD-1 response and spatial gene-expression annotation, respectively (19). We also generated matched bulk gene expression profiles (*i.e.*, RNA-seq) to benchmark the utility of ST to identify features of aPD-1 resistance. We uncovered the association of tumor-intrinsic cholesterol synthesis with anti-PD1 therapy resistance – an association that was not readily identified in our bulk profiling of matched tumor samples.

This observation aligns with recent studies linking cholesterol metabolism to immune regulation in various cancers, including melanoma, lung cancer, and breast cancer (51–54). However, multiple factors influence tumor cholesterol levels (53, 55), resulting in diverse immune phenotypes (51, 56–62). In this study, we further sought to understand whether cholesterol metabolism in tumor cells correlates with aPD-1 response, and which T cell evasion mechanism is associated with resistance (*e.g.*, recruitment vs. exclusion; activation vs. exhaustion). Our spatially aware gene expression analysis highlighted that tumor-intrinsic heterogeneity in cholesterol metabolism exists before aPD-1 treatment and is linked to therapy resistance, with T cell exclusion underlying the resistance phenotype in MC38.

## Results

### MC38 aPD-1 responders have elevated immune infiltration and activation

**Figure 1:**
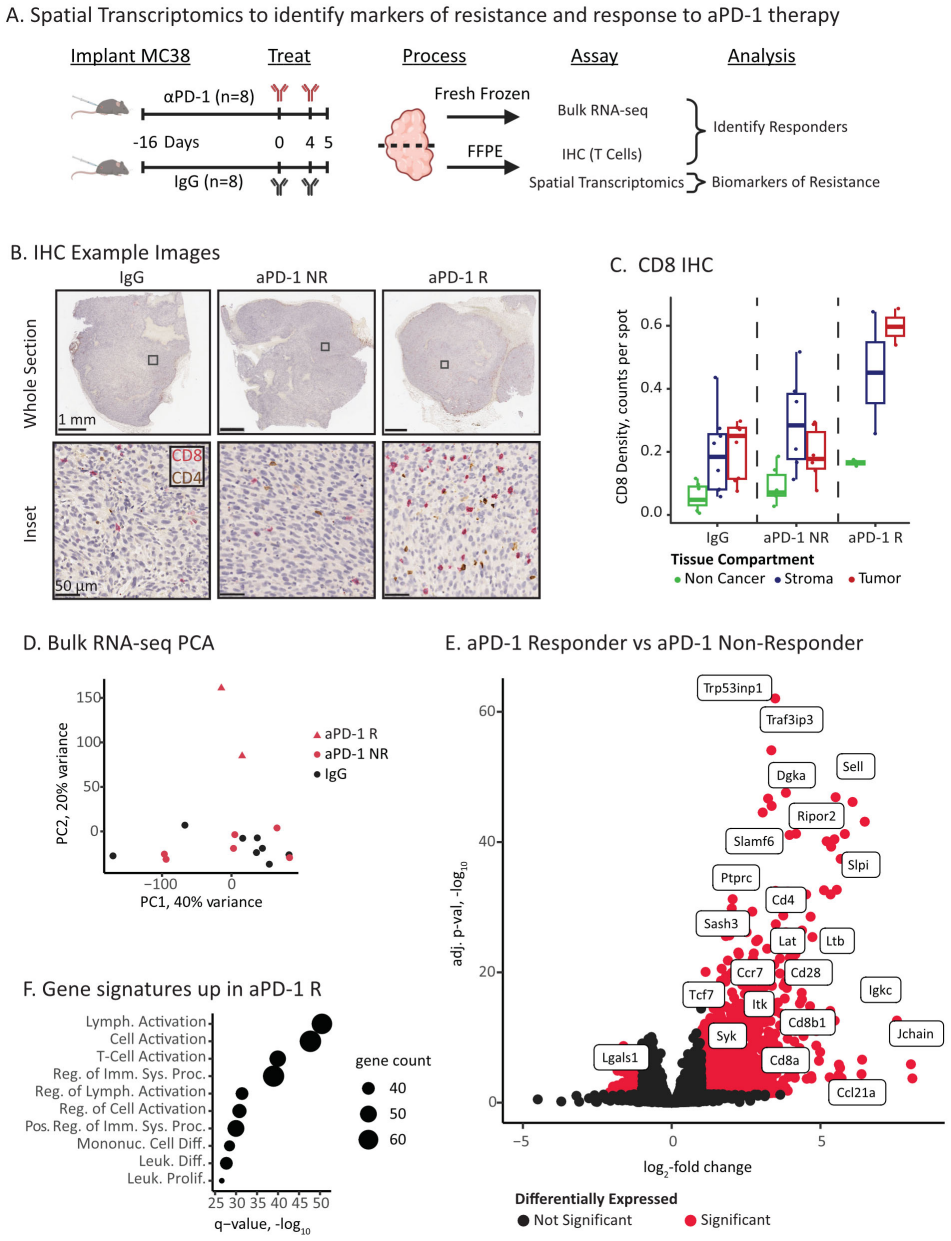
ICI responders have elevated immune infiltration and activation. **(A)** Experimental overview of ICI treatment and molecular characterizations. **(B)** Example IHC images of CD8 (red) and CD4 (brown) T cell markers. The columns correspond to treatment and response status: IgG control, aPD-1 Non-Responder (NR), and aPD-1 Responder (R). The top row displays the full tumor section, and the bottom row is a high resolution inset. Inset location is marked as a square in top row. **(C)** Quantification of CD8 positive cells in tissue compartments identified via digital pathology. IHC images were co-registered to the Visium H&E section, and positive cells in each capture spot were counted and normalized by the total number of spots per compartment and tumor section. **(D)** Principal components analysis (PCA) plot of bulk RNA-seq data for IgG control, aPD-1 non-responder (NR), and aPD-1 responder (R). **(E)** Volcano plot of differential gene expression between aPD-1 non-responder and aPD-1 responder tumors. Significant genes are marked in red and select immune related genes are labeled. **(F)** Gene set analysis (Fisher’s test) of the top significant genes upregulated in responders using Gene Ontology Biological Processes gene sets (109). Point size is proportional to significant genes count for each gene set.

MC38 is an MSI-H CRC model that has a variable response to aPD-1 therapy enabling the interrogation of response and resistance within the same model (28, 30, 41–44). MC38 tumors also recapitulate clinical disease features linked to therapeutic resistance, including T cell exhaustion and dense stroma (63). Here, we sought to understand tumor intrinsic determinants of response to aPD-1 by collecting treated MC38 tumors at an early pharmacodynamic time point (5 days post-treatment) to minimize confounding signal from tumor death, which would obscure transcriptional characterization of tumor cells (**Figure 1A**).

We first classified the aPD-1 treated tumors (n=8 IgG control and n=8 aPD-1) into responders and non-responders. Tumors were assessed by immunohistochemistry staining revealing a subset of tumors with elevated levels of tumor infiltrating CD8 T cells in the tumor compartments (**Figure 1B, C**, p-value = 0.034 - R vs NR Welch’s T-test). We designated these tumors as responders (n=2) (28). The other aPD-1 treated tumors had CD8 T cell levels comparable to the IgG baseline (**Figure 1C**), which we designated as non-responders (n=6; n=8 IgG).

We next assessed gene expression features of response and resistance using bulk RNA-sequencing. At a high level, the aPD-1 responder tumors were outliers compared to the aPD-1 non-responder and IgG tumors (**Figure 1D**). Consistent with the IHC, these differences primarily arose from immune populations that infiltrated the responder tumors, including increased expression of T cell (*e.g.*, Cd8a, Cd8b1, and Cd4) and immune activation markers (*e.g.*, Syk, Lat, Itk, Irf4) (**Figures 1E, F**, **Supplementary Table S1**). In contrast, non-responders had fewer significant genes (1024 vs. 94, respectively) and no significant enrichment of gene sets. Amongst these genes, we identified Lgals1 as a differentially expressed (DE) gene with an established immunosuppressive role (64, 65) (**Figure 1E**, **Supplementary Table S1**).

Overall, we were able to detect histology and expression-based hallmarks of ICI response at an early time point following aPD-1 administration. The two aPD-1 responder tumors demonstrated increased infiltration of activated cytotoxic T cells compared to non-responder and control tumors. However, bulk gene expression analysis did not provide substantial insights or associations with resistance.

### Spatial transcriptomic map of ICI-treated MC38 tumors

We sought to understand whether ST techniques could reveal resistance associations in non-responders that were missed by bulk profiling. To this end, we generated a transcriptome-wide spatial atlas across IgG control, aPD-1 responder, and aPD-1 non-responder tumors. ST was applied to the same tumors as profiled by bulk RNA-seq and histology.

**Figure 2:**
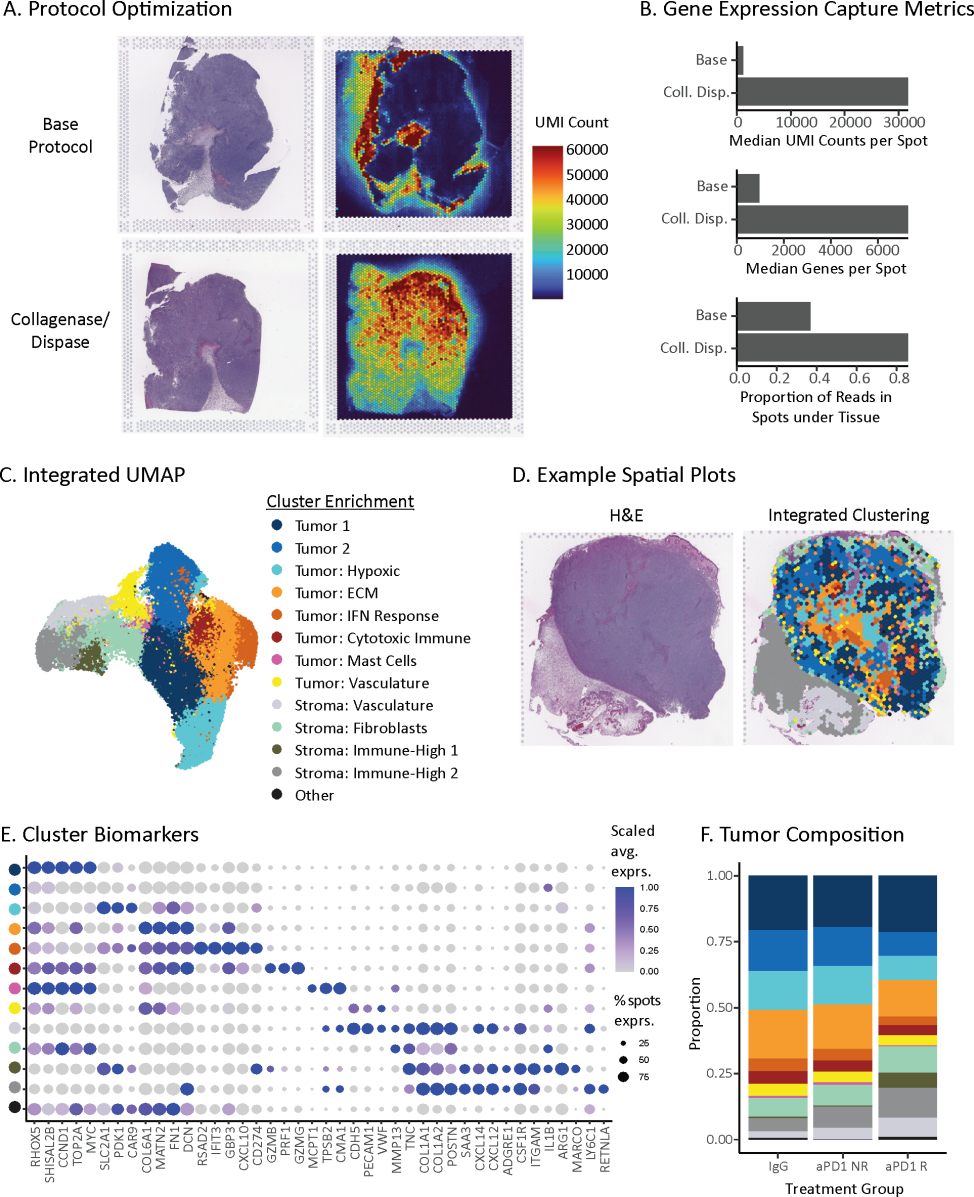
Spatial transcriptomic map of ICI-treated MC38 tumors. **(A)** Spatial distributions of unique molecular identifiers (UMIs) per spot with the base Visium protocol (top) or optimized protocol with collagenase and dispase permeabilization step (bottom). **(B)** Gene expression capture metrics of the base and optimized spatial transcriptomics protocols. **(C)** UMAP of unsupervised clusters from 48,636 ST spots across 21 tumor sections from 16 MC38 tumors. Clusters represent tumor and stroma subsets, named based on differentially expressed genes (**Supplementary Table S2**). **(D)** Spatial distribution of unsupervised clusters from **(C)** for a single tissue section (right) and its corresponding hematoxylin & eosin (H&E) stain (left). **(E)** Dot plot of differentially expressed biomarkers for each unsupervised cluster in the MC38 spatial atlas. Clusters are colored to match the legend above in **(C)**. Dot size is proportional to the number of spots that express the gene, and color matches the z-scaled gene expression. **(F)** Treatment and response group composition based on unsupervised clusters present within each tumor. Proportions are first averaged across replicate sections, if applicable, and then treatment group.

Initially, the ST base protocol (Visium for FFPE) yielded poor data quality (**Figures 2A, B**). The spots under the tissue exhibited a low number of unique molecular identifiers (UMIs) and genes detected with captured probes localized to spots outside of the tissue boundary (~63%) indicating capture of ambient signal beyond the tissue and suggesting inadequate tissue permeabilization. To address this issue, we added an additional enzymatic digestion step, which improved probe capture in spots under the tissue (methods). This modification resulted in a ~26-fold increase in median UMIs per spot and ~8-fold increase of median genes per spot (**Figures 2A, B**). This protocol optimization not only enabled analysis of these tissues but also offers a potential improvement for other challenging spatial transcriptomics indications.

Using the optimized protocol, we profiled 16 MC38 syngeneic tumors with 1-3 sections each for a total of 21 different sections. After stringent quality control filtering, our dataset consisted of 48,636 spots (55 µm diameter). Each spot captures low-bulk gene expression from an admixture of ~20 cells, which is small enough to enable the capture of dominant cell types in both mouse and human tumors (19). Given the low-bulk resolution, we refer to groupings of spots as subsets rather than single cells. Integrated clustering of the cohort partitioned the tissue into 8 tumor cell subsets and 4 stroma subsets observed across all treatment groups (**Figures 2C–E**, **Supplementary Table S2**). The tumor cell subsets stratified by three main types: First, tumor-dominant cell subsets (Tumor 1 and 2 clusters) lacking clear enrichment of other cell types, with Tumor 1 cluster characterized by greater expression of proliferation genes (*e.g.*, Ccnd1, Top2a, Myc). Second, tumor subsets enriched for microenvironment features, including hypoxia markers (*e.g.*, Slc2a1, Pdk1, Car9), extracellular matrix (ECM) genes (*e.g.*, Matn2, Fn1, Dcn, Col6a1), vasculature (*e.g.*, Cdh5, Pecam1, Vwf), and interferon response genes (*e.g.*, Rsad2, Ifit3, Gbp3, Cxcl10, Cd274). Third, tumor subsets enriched for immune markers, including cytotoxic immune genes (*e.g.*, Prf1, Gzmb, Gzmg) and mast cell protease genes (*e.g.*, Mcpt1, Cma1, Tpsb2). For stroma, we identified subsets enriched for vasculature genes (*e.g.* Cdh5, Pecam1, Vwf), fibroblast markers (*e.g.*, Col1a1, Col1a2, Postn), strong expression of secreted immune factors (*e.g.*, Saa3, Cxcl12 and Cxcl14) and myeloid markers (*e.g.*, Csf1r, Itgam, Cd163, Cd68). All subsets are named for these major features, respectively (**Figures 2C–E**, **Supplementary Table S2**).

The presence of tumor cell and stroma subsets across all tumors enabled us to assess whether the composition of tumors changed with treatment or response groups. Within the tumor cell compartment, the hypoxic tumor subset comprised a higher proportion of spots within aPD-1 non-responders compared to aPD-1 responders (14.3% vs. 8.9%, respectively) (**Figure 2F**). This observation is in line with hypoxic immune suppression reducing aPD-1 efficacy (66–68). Within the stromal compartment, the immune high-1 subset was more abundant in aPD-1 responders than non-responders (5.8% *vs*. 0.5%, respectively) (**Figure 2F**), which reflects an increased presence of immune cells following treatment. Overall, these differences in composition fit with a model where response to aPD-1 is associated with less hypoxia and more immune inflamed stroma (41, 69, 70).

### Cholesterol pathway associated with aPD-1 non-responders

**Figure 3:**
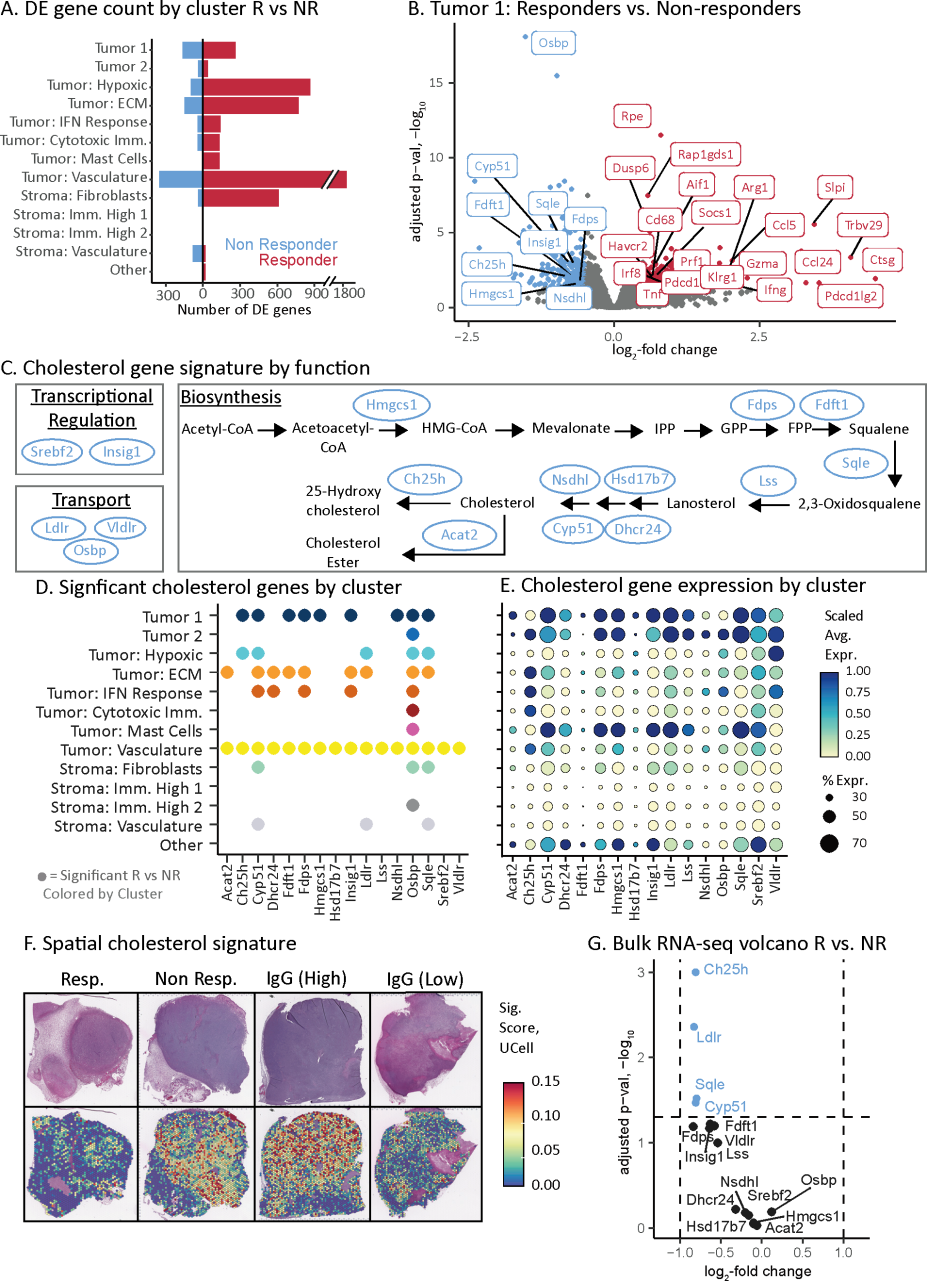
Cholesterol pathway associated with aPD-1 non-responders. **(A)** Transcriptome changes as assessed by number of differentially expressed genes across tumor and stroma subsets for the aPD-1 responders and non-responders. Double slash indicates a scale break used for data visualization. **(B)** Volcano plot of genes that are differentially expressed between aPD-1 responders and non-responders in the Tumor 1 subset. Selected genes are labeled with color indicating direction of expression change, consistent with **(A)**. **(C)** Pathway schematic of cholesterol regulation, synthesis, and transport. Genes identified via our spatial atlas as associated with non-responders are indicated (blue ovals). **(D)** Summary of cholesterol pathway genes that have significant expression changes associated with aPD-1 therapy resistance. Spatial atlas cell subsets are shown along the vertical axis. A dot is present if the gene is significantly upregulated in non-responders versus responders in that given subset. **(E)** Dot plot of cholesterol gene expression by subset. Dot size indicates the percent of spots in a subset that have expression of the given gene. Color represents the z-scaled average gene expression across the subset. The vertical axis labels from **(D)** extend to this figure. **(F)** Spatial gene expression plots of cholesterol pathway genes upregulated in non-responders depicted as a signature of 16 genes in **(C-E)** and calculated using UCell. Top row is H&E-stained section. Bottom row is the corresponding signature values. The two IgG samples show tumors with either high or low expression of the cholesterol gene signature. **(G)** Volcano plot of bulk RNA-seq data (from **Figure 1E**) depicting only cholesterol signature genes. Dashed lines indicate the significance cut-offs for log fold change and adjusted p-values. Blue dots indicate the genes that pass the threshold for significance by adjusted p-value.

Leveraging our spatial atlas, we sought to identify tumor intrinsic gene expression associated with aPD-1 response and resistance by performing differential expression analysis (methods) (71, 72) within the same tumor cell and stroma subsets across responder and non-responder tumors. Subsets enriched for tumor cells had substantially greater average number of DE genes than stroma subsets (520 vs 160 responders, 112 vs 32 non-responders) (**Figure 3A**, **Supplementary Table S3**) supporting our hypothesis that tumor cell intrinsic signaling underlies the variable response to aPD-1 treatment. Furthermore, responder tumors had more upregulated genes than non-responders, which is consistent with our bulk RNA-seq data indicating that most gene expression differences observed by bulk-RNA seq were driven by the tumor compartment.

We first evaluated response-associated DE genes within the tumor cell compartment that were upregulated in responders. The dominant signal across tumor cell subsets was driven by immune infiltration and inflammation (**Supplementary Table S3**). For example, in the Tumor 1 subset, upregulated genes included CD8 T cell (*e.g.*, Gzma, Prf1, Klrg1, Trbv29, Pdcd1, Havcr2) and macrophage/monocyte (*e.g.*, Cd68, Aif1) biomarkers (**Figure 3B**) (41). This subset also had upregulated interferon-gamma (73) and tumor necrosis factor signaling (74) in responders (**Figure 3B**). The concordance of these observations with established ICI biology (28, 41, 73, 74) supports the accuracy of our approach.

We next sought to identify potential tumor cell intrinsic mechanisms of resistance in non-responder tumors. The dominant signal in non-responders was higher expression of genes across the cholesterol pathway including transcriptional regulation, biosynthesis, and transport (**Figures 3B, C**). The upregulated DE genes in the Tumor 1 subset of non-responders included Hmgcs1, Nsdhl, Ch25h, Insig1, Fdps, Sqle, Fdft1, Cyp51, and Osbp (**Figure 3B**) (75–78). Across all tumor cell subsets, a total of 16 genes directly involved in cholesterol homeostasis were upregulated in non-responder tumors (**Figures 3C, D**). These cholesterol genes were more likely to be upregulated in tumor cell subsets than stromal ones (**Figures 3D, E**). The subsets that had the greatest number of upregulated cholesterol DE genes were Tumor 1 (9 genes), Tumor: vasculature (16 genes) and Tumor: ECM (9 genes) compared with lower abundance in Tumor: hypoxia (5 genes) and Tumor: IFN response (5 genes) (**Figure 3D**). These subsets comprised a higher proportion of the tumor clusters in responders and non-responders, respectively (**Figure 2F**). Taken together, these data indicate that cholesterol production was both upregulated in the tumor cells of non-responders, and that these cells comprised a higher fraction of the total in non-responder tumors.

We reasoned that if the level of cholesterol synthesis was due to intrinsic tumor cell heterogeneity, then the IgG control tumors should also have variable expression of cholesterol synthesis. Indeed, an IgG control tumor stood out by its low level of cholesterol gene expression comparable to responders (**Figure 3F**; **Supplementary Table S4**). These data indicate that the level of cholesterol synthesis is independent of aPD-1 treatment but is associated with the failure of aPD-1 to elicit T cell infiltration.

Finally, we assessed why bulk RNA-seq could not identify the association of cholesterol synthesis with non-responder tumors (**Figures 1A, E**) by evaluating all 16 cholesterol genes identified through ST (**Figure 3C**). Only 4 out of 16 genes (Ch25h, Ldlr, Sqle, and Cyp51) passed an adjusted p-value significance threshold (**Figure 3G**). However, these four genes had minor changes in gene expression between responders and non-responders (**Figure 3G**). Therefore, ST provided the necessary resolution and sensitivity to uncover the cholesterol synthesis pathway and revealed signals obscured in bulk profiling, providing novel insights into mechanisms of resistance to aPD-1 therapy in non-responder tumors.

### Location of cholesterol synthesis associated with dampened T cell response

We reasoned that the location of cytotoxic T cells and cholesterol synthesis should be incongruous if they lead to opposing ICI responses. To test this hypothesis, we leveraged the location of these two features independent of each other to identify *de novo* distance-based gene expression associations within aPD-1 responder and non-responder tumors. As a proof-of-concept for our approach, we first tested the relationship of cytotoxic T cells to the surrounding tumor because the role of T cells is well-established in the response of MC38 tumors to aPD-1 treatment (79). Then we tested the relationships of cholesterol synthesis to the broader tumor.

**Figure 4:**
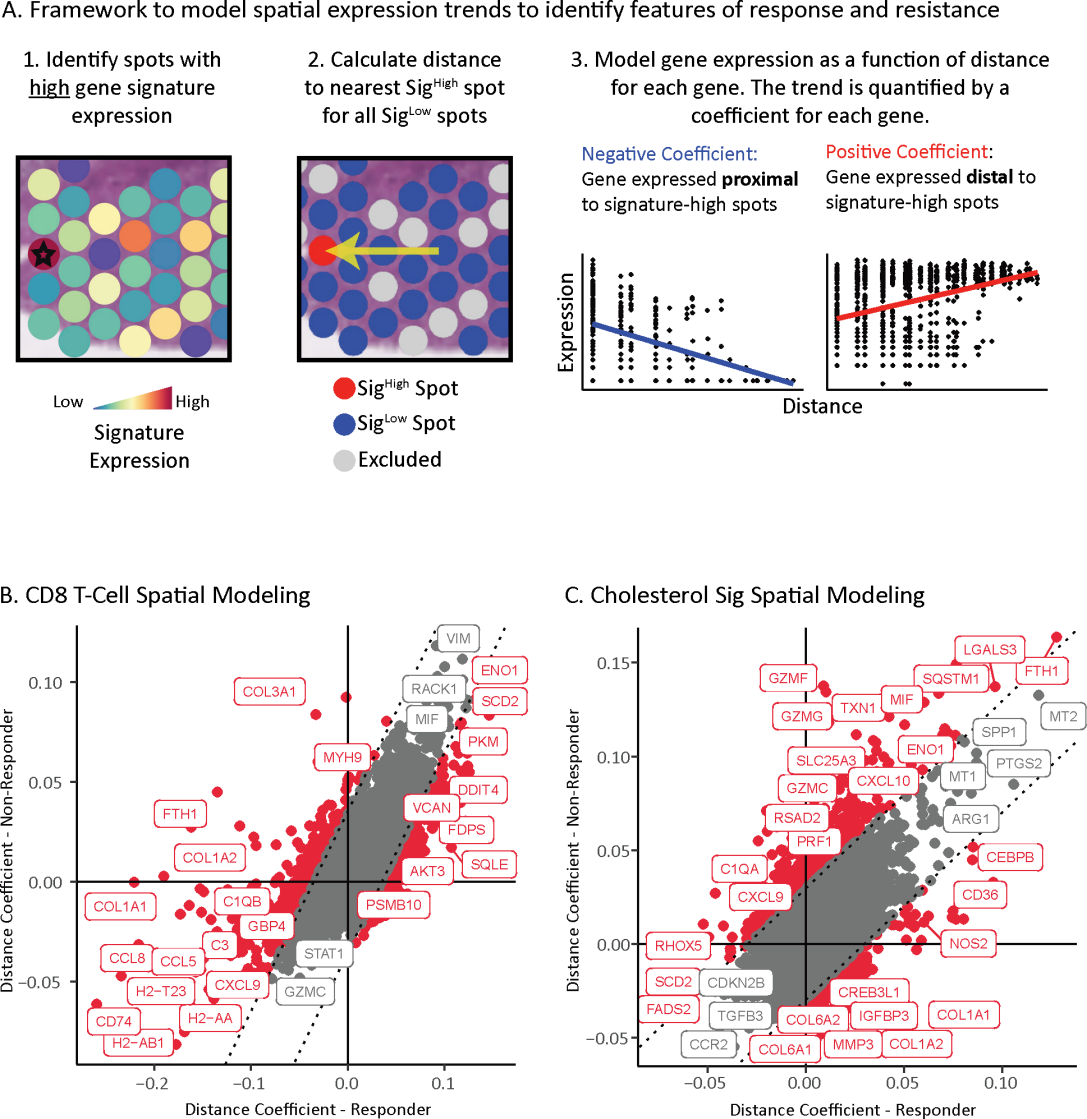
Location of cholesterol synthesis associated with dampened T cell response. **(A)** Framework to identify location-based changes in spatial gene expression that occur as a consequence of distance from a given feature of interest. Left panels: First, spots expressing high levels of the genes for a feature of interest are identified (indicated by star; Sig^high^). Next, spots with low expression of genes for the feature of interest are identified (Sig^low^). Non-tumor spots and spots with mid-signature expression are excluded from analysis to reduce confounding variables (grey spots). Distance from each Sig^high^ spot to the nearest Sig^low^ spot is calculated. Right panels: Representation of linear model used to identify gene expression of Sig^low^ spots as a function of distance from Sig^high^ spots. A coefficient is calculated for every gene to quantify the strength of the trend between expression and distance from a Sig^high^ spot. A positive coefficient indicates the expression increases with distance from a Sig^high^ spot, while a negative coefficient means the expression increases with proximity to a Sig^high^ spot. **(B)** Scatterplot of tumor spatial gene expression coefficients anchored on CD8 T cell Sig^high^ spots. Points are colored red if the difference between the coefficient in responders and non-responders is significant, and gray if it is not significant. The dotted lines indicate significance thresholds. **(C)** Scatterplot of tumor spatial gene expression coefficients anchored on cholesterol pathway Sig^high^ spots. Points are colored red if the difference between the coefficient in responders and non-responders is significant, and gray if it is not significant. The dotted lines indicate significance thresholds.

We developed a framework to identify gene expression changes that depend on their spatial proximity to a given feature – in this case, high gene signature score of either CD8 T cells or cholesterol synthesis in spots. This model quantifies the association between gene expression and the distance to the nearest given feature (*i.e.*, signature-high spot) for all spots that do not have that feature (*i.e.*, signature-low spots). We focused our analysis on tumor signaling, excluding spots from the stroma. We performed the modeling on a gene-by-gene basis, resulting in a coefficient for each gene that describes if the gene is expressed higher in tumor spots proximal to a feature (*e.g.*, T cells) yielding a negative coefficient, or distal from a feature yielding a positive coefficient (**Figure 4A**) (methods). We applied this framework separately to aPD-1 responder and non-responder tumors to understand the impact of response status on feature-based spatial expression patterns.

We first assessed the distance relationship of gene-expression relative to CD8 T cells. Antigen presentation genes spanning MHC-I and MHC-II (*e.g.*, H2-Ab1, H2-T23, H2-Aa, Cd74) and chemokines (*e.g.*, Ccl5, Ccl8, Cxcl9) were expressed closer to CD8 T cells in aPD-1 responder tumors than non-responder tumors (**Figure 4B**, **Supplementary Table S5**). Conversely, genes with positive coefficients that are expressed further away from CD8 T cells include hypoxia induced genes (*e.g.*, Eno1, Pkm, Ddit4) (80, 81), and cholesterol genes (*i.e.*, Sqle, Fdps) that were associated with ICI resistance identified in our prior analysis.

These distance associations between gene expression and CD8 T cells fits with well-established ICI biology. For example, the processing and presentation of antigens is a requirement to drive ICI efficacy through T cell activation (82–84). Likewise, among the chemokines, Cxcl9 is involved in recruiting and activating CD8 T cells, and its expression correlates with response to aPD-1 (85, 86). Conversely, hypoxia facilitates T cell exclusion and ICI resistance (87). These results support the validity of our approach.

Finally, we applied our distance framework to understand the impact of cholesterol-high regions on surrounding gene expression. Genes nearest the cholesterol-high regions in both responders and non-responders include the signaling ligand Tgfb3, which is associated with immunosuppression and drug resistance (88), and Ccr2, which is a myeloid marker associated with immune suppression (89, 90) and is upregulated by cholesterol (91)(**Figure 4C**, **Supplementary Table S6**). Interestingly, markers of cytotoxic T cell activity (*i.e.*, Prf1, Gzmf, Gzmg, Gzmc) (92–96) and inflammatory cytokines (*i.e.*, Cxcl10, Cxcl9) (85, 86, 97) were expressed further from cholesterol high regions in non-responders than in responders. The two spatial models support that high cholesterol in proximity to CD8 T cells dampens cytotoxic activity in MC38 non-responders.

## Discussion

In this study, we explored the features of response and resistance to aPD-1 therapy in MC38 tumors using spatial genomics. Our spatial atlas, validated through bulk RNA-seq and histology, identified tumors that either responded to immune checkpoint inhibitors with increased CD8 T cell infiltration and activation or failed to elicit an immune response. While all three datasets – ST, bulk RNA-seq, and histology – highlighted immune features of response, only the high-resolution ST atlas revealed strong associations with resistance. We found that non-responder tumor cells exhibited high expression of cholesterol synthesis genes compared to aPD-1 responders. Additionally, we demonstrated that cytotoxic T cells were excluded from cholesterol-rich regions in non-responders, suggesting that cholesterol or its derivatives play an immunosuppressive role that dampens tumor immunity. Of note, non-responders not only showed differential expression of cholesterol-related genes but also more distal expression of T cell activation markers from cholesterol-rich regions. Overall, these findings support that cholesterol metabolism is a tumor-intrinsic mechanism of resistance that impairs T cell activity and recruitment. Furthermore, we observe cholesterol metabolism heterogeneity in IgG control tumors indicating that this, at least in part, is an intrinsic resistance mechanism rather than an adaptive response to aPD-1 treatment.

Modulating cholesterol metabolism has been explored as a strategy to increase ICI efficacy. Pre-clinical studies have demonstrated that ICI combination with cholesterol modulating drugs improves response (59, 98–101). When combined with aPD-1 therapy, statins (*e.g.*, lovastatin) increase T cell infiltration in syngeneic lung cancer tumors resulting in reduced tumor growth (101). Likewise, in ARID1A mutant ovarian cancer models, simvastatin and atorvastatin elicit pyroptosis and synergize with anti-PD-L1 therapy (98). More generally, modulating lipid metabolism (*e.g.*, Wee1 inhibition) also improves ICI response (102); albeit we do not see association of other lipid pathways with aPD-1 response in MC38 tumors.

Cholesterol, its precursors, and its derivates are important mediators of immune cell function, supporting both activation and suppression. For example, cholesterol-enriched lipid rafts at the plasma membrane promotes T cell receptor (TCR) clustering for antigen recognition and subsequent cytotoxic function (56, 103). Then, within activated CD8 T cells, cholesterol biosynthesis and expression of its low-density lipoprotein receptor (LDLR) are necessary for proliferation (57, 58). Other cholesterol precursors are also essential for T cell activation (59) with their deficiency leading to an exhausted CD8 T cell phenotype (100, 104). Conversely, excess cholesterol can disrupt membrane lipid rafts precluding pro-inflammatory signal transduction (105, 106). Also, excess cholesterol can trigger cell stress resulting in T cell exhaustion (51, 60–62). This duality underscores the necessity for future studies to gain a deeper understanding of cholesterol’s role in immune regulation.

Overall, our study demonstrates an intriguing connection between tumor cell intrinsic cholesterol and T cell infiltration in the context of PD-1 therapy resistance. We identify a core set of cholesterol genes associated with this phenotype. Given the cellular complexity present within tumors, our study motivates further dissection of the interplay between cholesterol homeostasis and ICI response. More broadly, our study provides a proof-of-concept for the utility of spatial -omics technologies to identify putative therapy resistance mechanisms missed by bulk profiling.

## Methods

### MC38 cell culture

MC38 colon adenocarcinoma mouse cells were acquired from Dr. James Allison, MD Anderson Cancer Center, TX (RRID: CVCL_B288). The cells were tested for mycoplasma and STR profiling. MC38 cells were cultured in DMEM (ThermoFisher Scientific Cat# 11965092) Supplemented with 10% FBS (ThermoFisher Scientific Cat# 16000044), 10 mM HEPES (ThermoFisher Scientific Cat# 15630080), 1mM sodium pyruvate (ThermoFisher Scientific Cat# 1360070), 2 mM L-glutamine (Gibco), 1x MEM non-essential amino acids (ThermoFisher Scientific Cat# 11140050), and 1% pen/strep (ThermoFisher Scientific Cat# 15140122).

### Syngeneic tumor growth and treatment

Syngeneic tumors were grown by injecting 1 million MC38 cells subcutaneously in the right flank of 6–8-week-old female C57BL/6 mice (Charles River RRID: IMSR_JAX:000,664). Sixteen mice were enrolled for study if their tumor reached 150-250 mm^3^ on day 16. Mice were randomly assigned to control IgG or aPD-1 treatments (n=8 per treatment) and were dosed with 200ug, on days 16 and 20 (Q4Dx2). Mice were euthanized by CO_2_, and tumors were harvested on day 21 and stored in ice cold PBS before processing.

Each tumor was cut in half and processed by either FFPE or fresh-frozen tissue embedding. For fresh frozen blocks, the tumors were placed in cryomolds with ice-cold TissueTek O.C.T. Compound (VWR, 25608-930) on a pre-cooled aluminum block that was placed in a dry ice and ethanol mixture. Additional O.C.T. was added to ensure the entire tissue was covered. Blocks were stored sealed at -80°C. For FFPE blocks, the tumors were placed in 10% Neutral Buffered Formalin for 24-h fixation. The tumors were then processed for paraffin embedding in a Sakura VIP automated system with vacuum/pressure cycles, dehydrating in graded alcohols to xylene and then paraffin, and embedded into blocks for sectioning.

All mouse work was performed in accordance with Institutional Animal Care and Use Committees (IACUC) relevant guidelines at Charles River Laboratories and Bristol Myers Squibb under protocol number CR-0067.

### Bulk RNA-seq library preparation, sequencing, and analysis

Two sections measuring 100 microns of each fresh frozen tumor block were collected in microcentrifuge tubes. The tubes were kept on dry ice and shipped to Azenta Life Sciences (South Plainfield, New Jersey, USA) for downstream RNA sequencing. Total RNA was extracted from fresh frozen tissue samples using Qiagen RNeasy Plus Universal mini kit (Qiagen cat# 73404), followed by poly(A) enrichment using NEBNext Poly(A) mRNA Magnetic Isolation Module (New England Biolabs cat# E7490). Then strand-specific RNA sequencing library was prepared by using NEBNext Ultra II Directional RNA Library Prep Kit for Illumina (New England Biolabs cat# E7760S) following manufacturer’s instructions. Libraries were loaded on Illumina NovaSeq 6000 sequencer for a 2x150bp paired end reads.

Reads were aligned to the GRCm38 *Mus musculus* genome using STAR v2.6 and then quantified using RSEM v1.3.0. on Ensembl 91 annotated genes. Differential expression analysis between responders and non-responders was performed using DESeq2 (72). Genes were considered significant if the FDR adjusted p-value was less than 0.05 and the magnitude of the log2 fold-change was greater than 1 (**Supplementary Table S1**). Gene set analysis by Fisher’s test was performed using the 200 most significant genes higher in responders, and all 94 significant genes in non-responders by adjusted p-value (107, 108). Signatures from Gene Ontology: biological process set were used (109), and we report the top 10 most significant by FDR adjusted p-value.

### Spatial transcriptomics tissue staining, library preparation, and sequencing

The FFPE tumor blocks were sectioned 5 micron thin and placed on Visium gene expression slides (10X Genomics, 2000233). The tissues were dried, deparaffinized, stained by Hematoxylin and Eosin, and decrosslinked according to the manufacturer’s protocol (CG000409|Rev C), omitting the 85% ethanol deparaffinization step (Step 1.2.l). The H&E-stained slides were imaged on a Leica AT2 slide scanner using a 40x objective. Spatial transcriptomics libraries were generated with Visium for FFPE Gene Expression mouse reagents (10X Genomics #1000337) according to the manufacturer’s protocol (10x Genomics, CG000407|Rev D) with an added permeabilization step during RNA Digestion. A blend of collagenase B and dispase enzymes (EMD Millipore Sigma #SCR140) resuspended in Hank’s Buffered Salt Solution (ThermoFisher Scientific cat# 14175095) was added to the RNase buffer and enzyme mix to a concentration of 0.4 CDU/uL in step 3.1 to improve the permeabilization of the tissue. Additionally, after permeabilization at step 3.1.n, the slide was washed with 2X saline-sodium citrate buffer (Millipore Sigma S6639) with 0.1% sodium dodecyl sulfate (Millipore Sigma 71736).

Spatial transcriptomics libraries were sequenced according to manufacturer protocols (10x Genomics, CG000407 | Rev D) on an Illumina NovaSeq6000 using an S4 or S2 v1.5 flow cell or on a NextSeq2000 with a P3 flow cell with a read 2 length of 50 cycles.

### Digital pathology H&E image analysis

Deep learning neural networks (DenseNet V2) were trained to perform high-resolution semantic segmentation across the hematoxylin and eosin (H&E) images. Multiple models were organized in a class hierarchy to compartmentalize each model’s task to both improve segmentation performance and to simplify model training. These included five models to classify: staining artifacts, adipose tissue, necrosis, blood vessels, and TME (tumor stroma, non-cancer). Model training and deployment was done using HALO-AI v3.4 (Indica Labs). All model trainings used a transfer learning approach, leveraging models pretrained on large-scale natural image datasets (*e.g.*, ImageNet). Performance of the models was evaluated qualitatively via manual review of prediction overlays on top of images.

### Immunohistochemistry T cell phenotyping

A sequential 5 um section of the FFPE tumor blocks were cut for T cell dual immunohistochemistry. All steps were performed on a Lecia BOND RX stainer. Sections were stained first with Cd4 (Cell Signaling Technologies, 25229) and detected with Polymer Refine Detection (Lecia, DS9800). Sections were incubated in ER2 for 20 minutes. Then sections were stained with Cd8 (Cell Signaling Technologies, 98941) and detected with Polymer Refine Red Detection (Leica, DS9390). Finally, the slides were counterstained with hematoxylin and cover slipped for imaging on a Leica AT2 with a 40x objective.

Cell phenotyping was performed using HALO’s Multiplex IHC module v3.1.4 (Indica Labs). First, cells were segmented using the hematoxylin nuclear stain, and the stain intensity was quantified from the two chromogens. Next, a HALO AI Object Phenotyper model was trained to classify cells as expressing Cd4 or Cd8 in a supervised fashion by selecting several positive cells as well as hematoxylin-only stained cells. T cell density was quantified by summing the number of T cells that overlapped with co-registered Visium spots of each TME pathology class, then normalizing by sample by dividing the sum by the number of Visium spots per TME class.

### Tissue co-registration

The IHC and H&E images were co-registered to create a common coordinate system to transfer annotations between Visium spots, H&E class annotations, and T cell phenotypes. Tissue registration was performed using HALO v3.4 (Indica Labs) co-registration tool. The co-registration tool optimizes a B-spline transformation to optimally match whole-slide images. Image rotations and flips were applied to achieve rough alignment of IHC images to Visium images prior to co-registration. Co-registrations were performed first in an unsupervised manner. For co-registrations requiring improvement, multiple landmarks were annotated onto each image, manually identifying distinct tissue features conserved between the histological sections. Then the co-registration algorithm was redeployed in a semi-supervised manner where preference is given to transformation parameters that align landmark coordinates in addition to aligning image features. Finalized co-registrations were used to transform the spatial transcriptomic spots from the ST coordinate domain to the coordinate domain of the IHC image. Transformation was to the position of the spots including the individual spots’ height and width dimensions.

### Visium data QC, and unspecific probes

Sequencing data was processed using the 10X Space Ranger for alignment, and barcode and UMI counting. We applied SpotClean (110) to correct for mRNA diffusion where mRNA captured at a tissue spot originated from adjacent spots.

To assess probe specificity, we compared mean normalized pseudo-bulk gene expression levels between polyA-captured and probe-captured ST data derived from the same cohort. Linear regression was fit between two groups of expression values to identify outlier genes whose probe-based expression value deviated from fitted line by more than 3 standard deviations. These genes, considered to be captured by unspecific probes (**Supplementary Table S7**), were removed from downstream analysis.

Additionally, spots that located in too-light-to-be-tissue areas, with color values more than 1 standard deviation from the mean of all spots, were removed. Spots with less than 100 genes detected were removed. Finally, spots that were contiguous with at least 200 spots were retained for downstream analysis, ensuring that small, isolated tissue debris away from main tissue were excluded from downstream analysis.

### Cohort integration and clustering

Samples were first merged and normalized using SCTransform v2 (111). Spots with less than 2000 detected genes were filtered out before integration. Then, the integration was performed using the canonical correlation analysis implemented in Seurat R package (112) with number of anchor features set to 7000. Spots were clustered by the graph-based clustering approach of the same package. An optimal clustering resolution of 0.4 was selected manually with clustree-0.4.3 (113) library assisting in visualization.

### Marker gene identification and DEA

For each cluster, marker genes were identified using SCT assay through the *FindAllMarkers* function of Seurat (comparing each cluster to the rest of the spots). Resulting biomarkers were reported in **Supplementary Table S2** if they satisfied the following thresholds: 1) the percentage of cells where the gene is detected in was at least 10%, 2) absolute value of the log_2_ fold change was at least 0.25, 3) the negative log_10_ adjusted p-value was at least 2.

We conducted differential expression analysis between responders and non-responders for each cluster using a pseudobulk framework in DESeq2 (71, 72). Replicate sections were collapsed using the function *AggregateExpression*, and we considered genes significant if the adjusted p-value was less than 0.05 and absolute fold-change greater than 1.5.

### Modeling spatial gene expression trends around gene signatures

In this analysis, we first calculated signature scores of The *CD8_EarlyActiv* gene signature (114) and the cholesterol gene signature described in **Figure 3C** using the *AddModuleScore_UCell* (115) function from the UCell package. To determine whether each Visium spot is positive for a transcriptional signature, we used the distribution of the signature across the whole tissue section. A spot was considered positive for a signature if its score was greater than or equal to the median plus 1.5 standard deviations of the signature distribution. A spot was considered negative for a signature if its score was less than or equal to the median. Other spots between these cut offs were excluded from analysis.

We employed linear models to identify genes associated with proximity to signature high regions in responders and non-responders separately. The analysis was restricted to tumor spots located within ten spot lengths from a signature high spot. Stroma and non-cancer spots called by digital pathology annotations were excluded. In our models, both the sample ID and the signature score were included as covariates. Signature scores were adjusted to account for transcripts of signature genes that diffused from the signature high region. We considered a gene to be significant if the Bonferroni adjusted P-value was less than 0.05, and the estimated coefficient was greater than the mean of the coefficients plus three standard deviations. To identify outliers in gene expression estimates between responders and non-responders, we first calculated the difference in estimates between two groups for each gene. We then calculated the mean and standard deviation of these differences. Genes with differences greater than two standard deviations from the mean were considered outliers and called significant.

## Data Availability

The datasets presented in this study can be found in online repositories. The names of the repository/repositories and accession number(s) can be found below: https://www.ncbi.nlm.nih.gov/geo/, GSE284264, https://www.ncbi.nlm.nih.gov/geo/, GSE284989.
