# Supplementary material for: Spatial genomics reveals cholesterol metabolism as a key factor in colorectal cancer immunotherapy resistance
Source: Front Oncol. 2025 Mar 18;15:1549237. doi: 10.3389/fonc.2025.1549237 (PMC11959564; doi:10.3389/fonc.2025.1549237)
Supplement: Supplementary file 1 [file DataSheet1.docx]

Supplementary Material

# Supplementary Data Captions

The following supplementary data tables are included as part of this article in excel files.

Supplementary Table S1: Bulk RNA-seq differential expression. Each tab of the workbook includes the results from 3 different comparisons between treatment groups.

Supplementary Table S2: Spatial transcriptomics cluster biomarkers. Lists differentially the expressed genes and statistics for unsupervised clustering analysis.

Supplementary Table S3: Spatial transcriptomics differential expression between responders and non-responders. Includes a different tab for every cluster.

Supplementary Table S4: Cholesterol signature heterogeneity. Values are averaged UCell signature scores, by cluster in the different tumors.

Supplementary Table S5: CD8 T cell spatial modeling statistics. Lists the distance from CD8 T cell signature coefficients for each gene responders and non-responders.

Supplementary Table S6: Cholesterol signature spatial modeling. Lists the distance from cholesterol signature coefficients for each gene responders and non-responders.

Supplementary Table S7: Unspecific probes. List of probes in Visium for FFPE that were removed from analysis due to showing unspecific hybridization.
